# Predicting MHC I restricted T cell epitopes in mice with NAP-CNB, a novel online tool

**DOI:** 10.1038/s41598-021-89927-5

**Published:** 2021-05-24

**Authors:** Carlos Wert-Carvajal, Rubén Sánchez-García, José R Macías, Rebeca Sanz-Pamplona, Almudena Méndez Pérez, Ramon Alemany, Esteban Veiga, Carlos Óscar S. Sorzano, Arrate Muñoz-Barrutia

**Affiliations:** 1 Centro Nacional de Biotecnología, Consejo Superior de Investigaciones Científicas, 28049 Madrid, Spain; 2 Departamento de Bioingenieria e Ingenieria Aeroespacial, Universidad Carlos III de Madrid, 28911 Leganés, Spain; 3 Bioengineering Department, Imperial College London, London, SW7 2AZ UK; 4 Unit of Biomarkers and Susceptibility, Oncology Data Analytics Program (ODAP), Catalan Institute of Oncology (ICO), Oncobell Program, Bellvitge Biomedical Research Institute (IDIBELL), 08908 L’Hospitalet de Llobregat, Spain; 5 Centro De Investigación Biomédica en Red de Epidemiología y Salud Pública (CIBERESP), Madrid, Spain; 6 Procure Program, Institut Català d’Oncologia- Oncobell Program, Catalan Institute of Oncology (ICO), Oncobell Program, Bellvitge Biomedical Research Institute (IDIBELL), 08908 L’Hospitalet de Llobregat, Spain; 7 Instituto de Investigación Sanitaria Gregorio Marañón (IiSGM), 28007 Madrid, Spain

**Keywords:** Computational biology and bioinformatics, Computational platforms and environments, Genome informatics, Machine learning, Protein analysis, Protein function predictions, Software, Tumour immunology, Tumour immunology

## Abstract

Lack of a dedicated integrated pipeline for neoantigen discovery in mice hinders cancer immunotherapy research. Novel sequential approaches through recurrent neural networks can improve the accuracy of T-cell epitope binding affinity predictions in mice, and a simplified variant selection process can reduce operational requirements. We have developed a web server tool (NAP-CNB) for a full and automatic pipeline based on recurrent neural networks, to predict putative neoantigens from tumoral RNA sequencing reads. The developed software can estimate H-2 peptide ligands, with an AUC comparable or superior to state-of-the-art methods, directly from tumor samples. As a proof-of-concept, we used the B16 melanoma model to test the system’s predictive capabilities, and we report its putative neoantigens. NAP-CNB web server is freely available at http://biocomp.cnb.csic.es/NeoantigensApp/ with scripts and datasets accessible through the download section.

## Introduction

Cancer cells can accumulate many mutations that change protein sequences. It can lead to MHC-restricted T-cell epitopes^1^. Identifying the tumor-specific epitopes that elicit T cell cytotoxic responses represents a major challenge for cancer immunotherapy, particularly to design personalized therapies^1,2^. Finding neoantigens in every cancer patient will be fundamental for the next generation of antitumor immunotherapies.

A plethora of neoantigen discovery pipelines has been described to enable the prediction of epitopes from genetic information. However, current pipelines are human-centered and, thus, are primarily designed for clinical usage^3,4^. Among the preeminent research lines, genomic analysis adjustments^3,5–8^, and neoepitope ranking practices^5,6,8,9^ have been prioritized over affinity binding or immunogenicity prediction algorithms. Despite this, the latter ones remain a critical component of the overall workflow for which limited available options exist^10^.

The absence of dedicated tools for the alternative in vivo mouse models hinders pre-clinical cancer immunotherapy research. Hence, laboratories have to produce or adapt to ad-hoc human pipelines. The pipelines Epi-Seq^11^, pVAC-Seq^3^, MuPeXI^9,12^ and Neoantimon^13^ offer modified versions for the murine model. These platforms follow the canonical prediction process, based on sequencing data to estimate the gene expression and the predicted affinity with the T-cell receptor (TCR) of the mutated peptide^10^, which is a prerequisite to elicit an immune response^1^. Epi-Seq performs a full-analysis from DNA and RNA reads file, however, it is not tailored for neoantigen detection, as it was conceived for the discovery of common tumor antigens. The other platforms lack genome preprocessing and variant calling in its analysis. Hence, in these three options, a variant call format file (VCF) its needed for its usage. Among them, solely MuPeXI is accesible as a webserver whilst pVAC-Seq and Neoantimon have to be installed locally and require a BAM file to estimate the levels of gene expression, which underscores the importance of a comprehensive and integral pipeline as a freely accessible webservice.

The algorithms underpinning the prediction of immune response differ aming these options. Epi-Seq and MuPeXI use NetMHCPan^14^ and its pan-specific variant, NetH2pan^15^, which rely on dense neural networks for binding affinity prediction. These tools have been trained with samples from the major histocompatibility complex (MHC) of mice or H-2. pVAC-Seq and Neoantimon also include MHCflurry^16^, which recently has been upgrated to include an estimation of immunogenicity through an antigen processing model using a convolutional neural network. In general, among the supervised machine learning methods that have facilitated the identification of neoepitopes, artificial neural networks have proven to be highly efficient^17^. However, recurrent neural networks (RNN) remain quite unexplored even if they are better suited for sequential problems, as attested by their extensive usage in natural language processing systems^18^. As a case, long short-term memory (LSTM) units are, at present, used for protein prediction of function and interactions^19,20^.

Prediction models have relied on gene expression information from tumor samples to determine putative peptides for intervention^1^. However, current approaches depend on genetic information from DNA sequencing to determine mutations^5,8^. This dependence hinders temporal performance and increases intervention costs, but whole-exome sequencing (WES) is justified for its improved selectivity^21^. Hence, a system may rely exclusively on RNA sequencing (RNA-Seq) to simultaneously identify mutations and gene expression levels^21^. If compensatory methods in neoepitope prediction are present, a tool designed for pre-clinical use may only rely on mutational information from RNA-Seq for a cost-effective solution. We developed an integrated pipeline optimized for a murine model that finds putative neoepitope via next-generation sequencing (NGS) tumor variant calling and ranks them using LSTMs. This novel platform is only based on RNA-Seq, and is automated for a given haplotype. As a proof-of-concept, we trained our system with the H-2K^b^ haplotype (MHC class I) to be tested for the commonly used B16 melanoma model in C57BL/6 mice, but the tool is compatible with additional typings that correspond to the most common in C57BL/6^22^ and FVB/NJ^23,24^.

Furthermore, the NAP-CNB is available separately as sequence affinity binding predictor. Entries are also constrained by a minimum length for each haplotype as tool is conceived for a NGS-based analysis in which proteins are submitted in their full extension. The resource NAP-CNB is freely available as a web server at http://biocomp.cnb.csic.es/NeoantigensApp/.

## Methods

**Figure 1:**
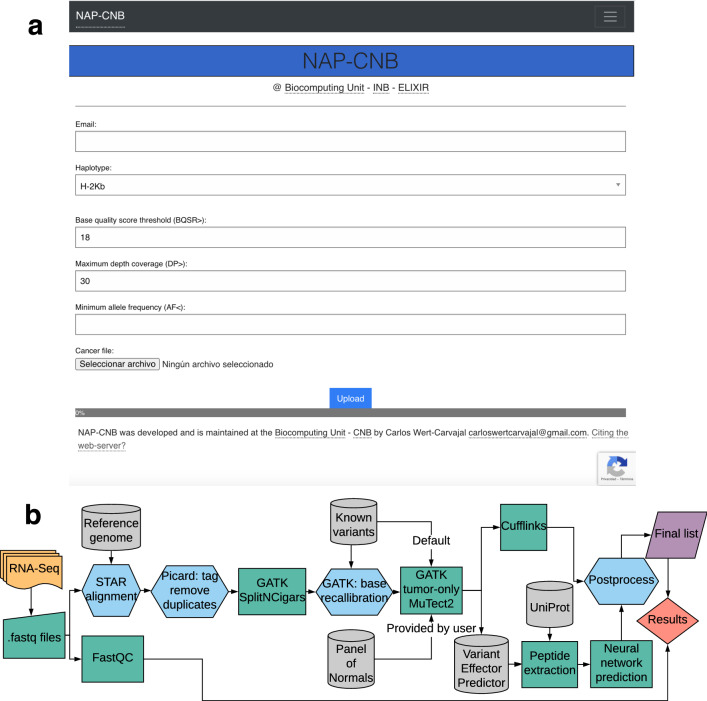
Workflow for the integrated pipeline. (**a**) The user interface of NAP-CNB with the fields required for NGS analysis. Users can introduce filters of GATK for base quality score recallibration (BQSR) of RNA-Seq reads, minimum depth coverage (DP) and allele frequency (AF). Additionally, users may submit peptidic sequences for affinity prediction. Individual submissions are haplotype-specific, and results are sent to an email address. (**b**) Workflow for the integrated pipeline. Firstly, the sample is preprocessed before variant calling. Quality control through FastQC and STAR alignment with the reference genome is followed with protocols from Best Practices of GATK. Known variants are introduced through known polymorphisms or a panel-of-normals if requested, andsufficient non-tumor RNA-Seq reads are provided. MuTect2 is used for variant calling, and plausible single nucleotide variant (SNV) mutations translated into peptidic sequences for prediction with the RNN model. Gene expression is quantified through Cuffquant in Cufflinks.

The proposed pipeline employs genome preprocessing tools, variant calling software, and customized neural network architecture to obtain putative neoantigens from RNA-Seq experiments. As an integrative tool, the workflow has been adapted into a web server for RNA-Seq file submissions with filtering options available at the preprocessing level, as shown in Fig. 1a. A tumor RNA-Seq file should be inputted as “.fastq.gz” together with the MHC class I type and an email address to receive the final results in less than ten hours. The binding affinity predictor is also available separately to be used for peptides sequences in FASTA format, which is able to process 5000 sequences in less than 30 seconds.

### Variant calling: from RNA-Seq to mutant peptides

The somatic mutations suitable for neoantigen prediction are obtained from the gene expression of tumor tissue (RNA-Seq). NGS technologies that produce a FASTQ file are required for this protocol.

First, a quality assessment report is produced using FastQC (v0.11.8)^25^ for user evaluation. In terms of preprocessing, the RNA-Seq file is realigned with a reference genome for further processing with STAR (v2.6.0a)^26^. The resulting BAM file is processed with Picard (v2.19.2)^27^ for further refinements such as annotation and duplicate marking. Subsequently, Genome Analysis Toolkit (GATK, v4.1.2.0)^28^ is used for exon segmentation, through the “SplitNCigarsReads” protocol, and base quality score recalibration (BQSR) following Best Practices guidelines^29^. As indicated in Fig. 1b, this part serves as a preprocessing of the RNA-Seq reads *per se* before variant calling. At this level, the user may introduce more flexible or conservative restrictions at the quality level by modifying the default threshold of BQSR.

The MuTect2 variant caller^30^ from the GATK package is used in its tumor-only mode (Fig. 1b), which is computationally less expensive but provides a higher number of false positives^31^. Even if designed primarily for DNA-Seq reads, MuTect2 has shown to be efficient in calling mutations from RNA-Seq^32^. By default, tumoral RNA-Seq is matched with databases of single nucleotide polymorphisms (dbSNP), although it can be used with a panel-of-normals (PoN) by construction. Following depth coverage (DP) filtering, the variants are submitted to Variant Effector Predictor (VEP) from Ensembl (v100.0)^33^ for annotation and extraction of mutant peptide sequences identified as missense variants. An additional allele frequency (AF) can be introduced at submission. Finally, a script matches the resulting UniParc reference from VEP to extracted UniProt proteins for protein-level prediction^34^.

Additionally, Cufflinks (v2.2.1)^35^ is used for mRNA abundance estimation as measured by fragments per kilobase million (FPKM). As there is no range for optimal neoantigen expression, this metric is provided to the user for its examination (Fig. 1b).

Hence, NAP-CNB provides a simplified interface for users to submit neoepitope prediction jobs to a webserver. Hence, it removes the need for a local machine, as required by Epi-Seq^11^, pVAC-Seq^3^ and Neoantimon^13^ and, in contrast with MuPeXI^9,12^, it additionally provides variant calling capabilities. Nonetheless, current customization remains limited. The output consists of a list of sequences with a softmax score and a complementary binary metric from postprocessing. Additionally, levels of expression are also included for the user. Jobs can be downloaded as lists or “.csv” files, which permits easy analysis and compatibility with data analysis software to perform further candidate sorting and selection.

### Dataset generation and preprocessing

Sequences of MHC-I binding peptides were obtained from the IEDB database^36^ for the H-2D^b^, H-2D^d^, H-2D^q^, H-2K^b^, H-2K^d^, H-2K^q^, H-2L^d^ and H-2L^q^ haplotypes, although here we present the procedure and results of H-2K^b^ as a case. Given the different binding assessment methodologies considered in IEDB, elements were binarized by their MHC class I classification as positive or negative, per IEDB standards. The datasets, by entries accession number, are available at NAP-CNB.

Firstly, peptides deemed as antigenic were processed to extract their binding sites. These correspond to positive epitopes from IEDB as classified by their qualitative labels “Positive High”, “Positive Intermediate” and “Positive Low” for each MHC class I haplotype in mice irrespective of the assay type. A further selection criteria was to include only epitopes with protein identifications to generate negatives and resize the sequence to a given length. Consequently, sequences were aligned with its protein source through the Smith-Waterman algorithm^37^ to obtain the remaining sequence as negative samples (Suppl. Fig. 1). Additionally, epitope regions were extended through the original sequence to have a regular size (Suppl. Fig. 1). In contrast with previous methods, a given prevalence (i.e., the fraction of the minority class) was not imposed on the dataset. In total, for H-2K^b^, 4,828 peptide entries were processed into 251,049 sequences with 6714 positive entries and 244,225 negatives. A 10% split was used for test set generation. Concerning blind test data, IEDB datasets 1034799 and 1035276 were processed through the previous procedure and by the method described by^15^. Additional information concerning the dataset for each haplotype is available in the download section of NAP-CNB.

Further postprocessing was implemented with a majority vote algorithm that considered mutations to the most similar amino acid, given by the BLOSUM62 matrix^38^, for each position. In other terms, a sequence modified its classification if there was a consensus among its most akin peptides.

### Neural network training

The neural networks were implemented through Keras (v2.2.4)^39^ and TensorFlow (v1.11.0)^40^. A scalable routine was used for architecture optimization through simplified datasets (Suppl. Fig. 1) until one competent was obtained. Moreover, training was done with “on-batch” class balancing and data augmentation. The latter increased the number of positives sequences through random substitution of a given number of amino acids with similar ones from the BLOSUM62 matrix^38^, with a given tolerance (Suppl. Fig. 3). The training was performed through fivefold cross-validation, for hyperparameters tuning and optimization of balancing and augmentation, generating a total of 80 models for the actual dataset.

The initial toy model was used for embedding selection and tuning of neural architectures (Suppl. Table 1A,B), which was maintained in the type and depth of layers in later configurations. At this stage, there were no significant improvement in any of three low-dimensional embeddings^41–43^, against a one-hot encoding (Suppl. Table 1A). Hence, we maintained the dimensions given by the naturally occurring amino acids. While an intermediate dataset (Suppl. Fig. 1C) was introduced for data balancing and augmentation. The final model was produced with the complete dataset and cross-validation of the number of internal LSTM units at each layer, the number of on-batch sequence augmentations, and its tolerance, and the on-batch class balancing.

**Figure 2:**
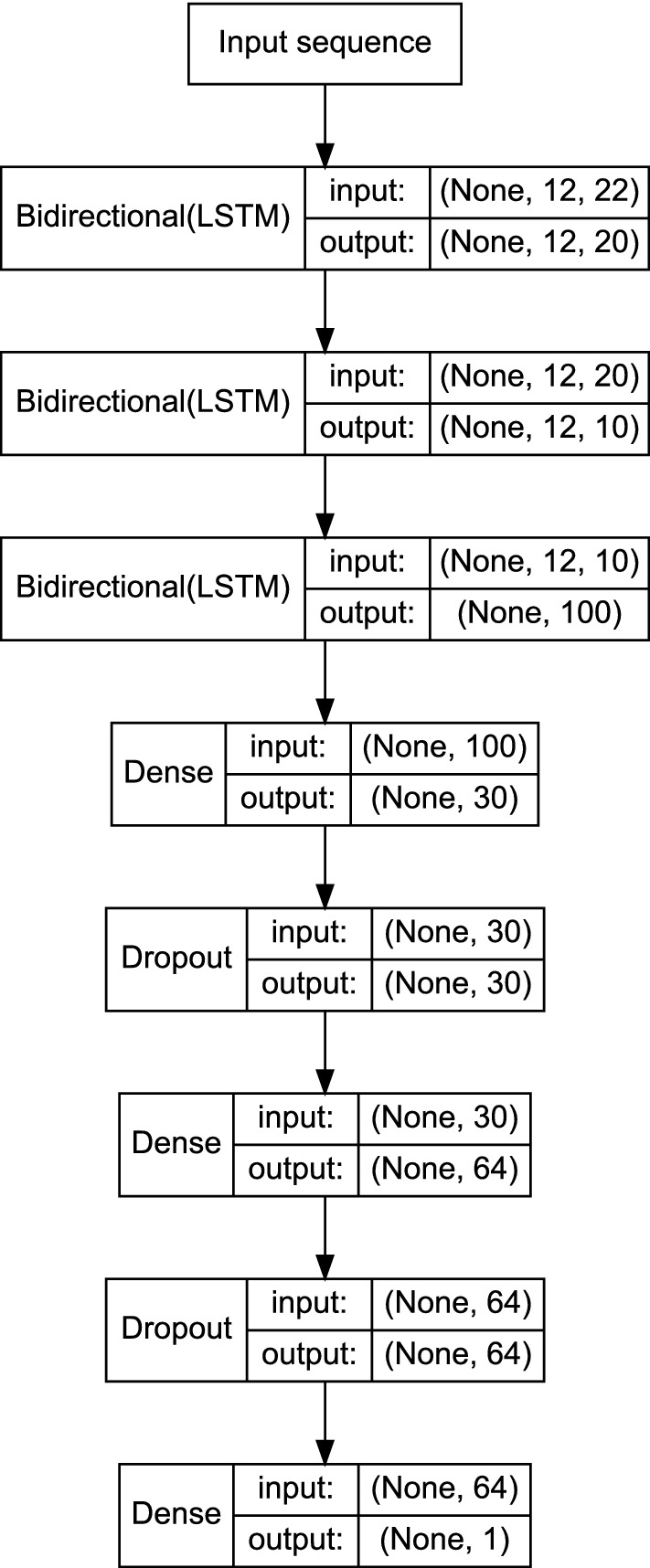
Neural network model of the binding affinity prediction for H-2K^b^. The input sequence corresponds to a one-hot encoding of a 12 mer peptide sequence extracted from the preprocessing workflow. The number of LSTM units corresponds to the input sequence’s overall length across the three consecutive layers. Following the RNN, two hidden dense units, with alternating dropouts, serve to process an affinity probability.

In the final architecture, peptide sequences of a given length are introduced with a one-hot encoding representation to three consecutive bidirectional LSTM layers, followed by three layers of dense neurons with two intermediate dropouts units. The output layer consists of a dense neuron, with a soft-max activation, which yields the affinity estimation probability. The overall network is represented in Fig. 2.

**Table 1:** Binary classification metrics for the final fivefold cross-validated algorithm for the H-2K^b^ typing.

AUC ROC	ACC	PPV	Sensitivity	Specificity	F1
(±SD)	(±SD)	(±SD)	(±SD)	(±SD)	(±SD)
$0.95\pm 0.04$	$0.977\pm 0.004$	$0.6\pm 0.1$	$0.62\pm 0.09$	$0.988\pm 0.004$	$0.6\pm 0.1$

The reported mean statistics estimators correspond to AUC ROC, accuracy (ACC), precision or positive predictive value (PPV), and sensitivity and specificity with their harmonic average (F1). The prevalence of positive samples was around 1:40.

### Sequencing raw data

An in vitro B16 melanoma cell line with a H-2K^b^ haplotype was processed for RNA extraction and sequenced through an NGS Illumina HiSeq2000. From the FastQC analysis, all evaluated parameters were satisfactory except from the presentation of four over-represented sequences corresponding to Illumina single end PCR primer and technical noise as TrueSeq adaptors. Trimming of these sequences was done before RNA-Seq processing. The resulting “.fastq.gz” file was introduced for analysis in a local server.

## Results

### Cross-validation metrics

**Figure 3:**
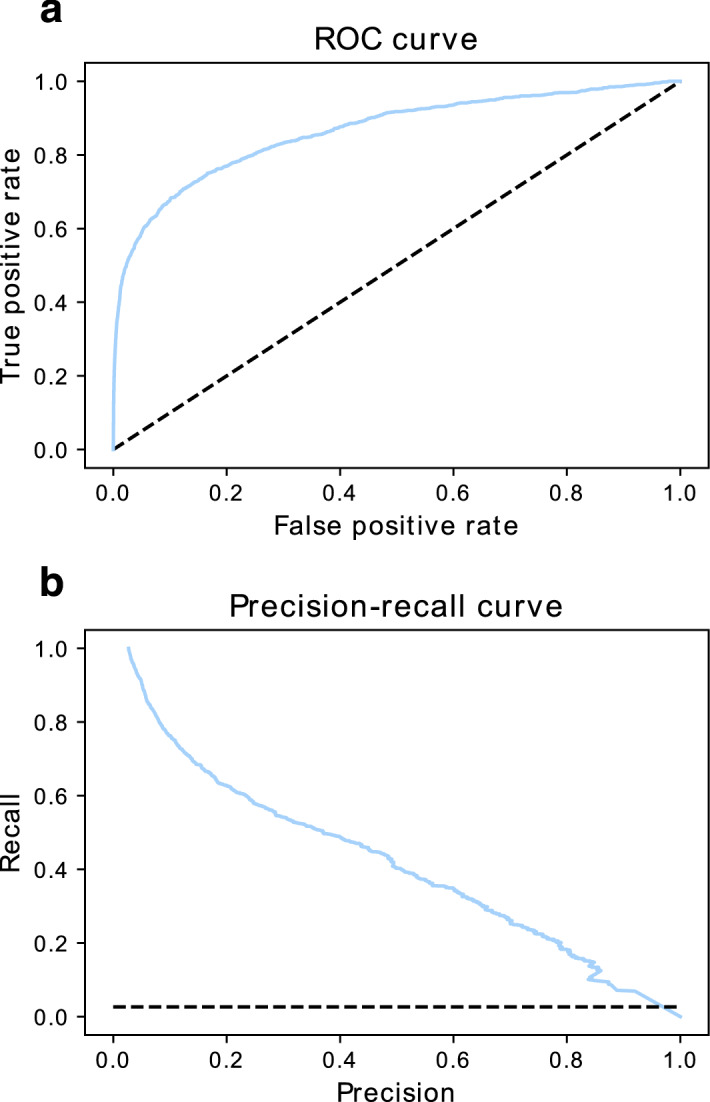
ROC and precision-recall curves for the final model trained with H-2K^b^ samples. (**a**) ROC curve for 10% test partition with an AUC of 86.5%, the dashed line shows chance level. (**b**) Precision-recall curve with the prevalence of around 3% shown as chance. The precision-recall AUC is 41.97%, whereas a random guess corresponds to an AUC of 2.64% for the same data imbalance.

**Figure 4:**
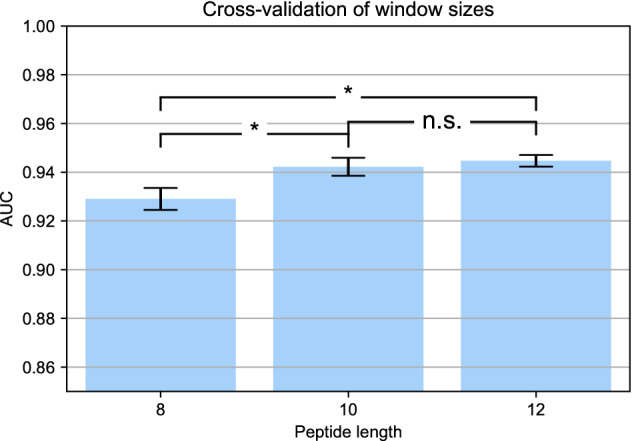
Cross-validation of peptide window sizes for H-2K^b^. The area under the curve of the receiver operating characteristic curve using 8 mers, 9 mers, and 12 mers obtained through fivefold cross-validation in different conditions. The windows are obtained from the mutated peptide sequence centered at the location of the SNV. Significant differences between means (Student’s t-test, p $<0.05$) are shown.

Initial architectures, based on LSTM and dense layers, showed performance improvements, in terms of the area under the curve for the receiver operator characteristic (AUC ROC), for higher depth models (Suppl. Table 1A). Despite this, these changes did not have an impact as significant as “on-batch” balancing and data augmentation. In particular, modifications of a “virtual” prevalence raised AUC ROC and F-1 values to 20% in test sets (Suppl. Table 1C) and decreased the degree of overfitting. All parameters were adjusted through grid search on the final model under a limited number of epochs (see Additional file 2—Grid search parametrization). As observed in Table 1, the network’s final AUC ROC for H-2K^b^ reached 95%, albeit with an acceptable F1 score, due to the assumed low prevalence. The complete cross-validation results of each model are available at NAP-CNB. For further evaluation in the H-2K^b^ haplotype, 10% of the original dataset was used as a test set of the selected parametrized system. In Fig. 3, both the ROC and the precision-recall curve are shown. The latter reflects how the system fares against a high-class imbalance. In terms of metrics, the ROC AUC for the test sample was 86.5% with 97.2% accuracy. Notwithstanding, the proposed ensemble method for postprocessing could increase precision by 7.6%. Throughout cross-validated models, window sizes of 8, 10, and 12 amino acids were tested for predictive performance. Sequences of 12 amino acids produced more accurate models (Fig. 4). This result may indicate that antigenic determinants are not sufficient for peptide classification and distal amino acids carry additional predictive information. The distribution of sequences classified as positive and a sensitivity analysis from random classifications showed similar results (Suppl. Fig. 4). In contrast, NetH2pan has reported a greater accuracy for short sequences around epitopes^15^.

**Table 2:** AUC ROC scores and minimum required peptide lengths of haplotypes implemented in NAP-CNB.

Haplotype	AUC ROC(±SD)	Peptide length (mer)
H-2D^b^	$0.7\pm 0.1$	12
H-2D^d^	$0.9\pm 0.1$	12
H-2D^q^	$0.8\pm 0.1$	12
H-2K^k^	$0.96\pm 0.06$	8
H-2K^q^	$0.9\pm 0.2$	12
H-2L^d^	$0.9\pm 0.1$	12
H-2L^q^	$0.7\pm 0.2$	8

The AUC ROC corresponds to the fivefold cross-validation average of the best configuration obtained through grid-search parametrization. In all haplotypes 128 models were initially generated for lengths of 8, 10 and 12 amino acids with additional fine-tuning for some instances.

The cross-validation metrics of the all generated haplotypes presents both enhancements and reductions in efficacy, as shown in Table 2. In the typings H-2K^d^, H-2K^k^ and H-2L^q^ the best performance corresponded to 8-mers. We provide, as an example of further benchmarking and binary metrics, additional results for H-2K^d^ (Suppl. Material. H2-Kd). Moreover, for this typing, we report a suboptimal cross-prediction with H-2K^b^ (Suppl. Material. H2-Kd), which evidences the need for individual networks for each haplotype.

### Benchmarking

In contrast with NetH2pan^15^, which is the benchmark used for MHC class I affinity prediction in mice, the reported cross-validated AUC ROC, in Table 2, were comparable or superior with a 95% for H-2K^b^, which is 3% higher, and a similar performance in PPV. Results vary for each haplotype and we report a hindered efficiency in some haplotypes such as H-2D^b^. Results of binding affinity are also on par with those from MHCflurry 2.0^16^, showing improved scores for H-2K^k^ and a worsening for H-2L^q^, for instance. MHCflurry 2.0 does provide a more refined metric for immunogenicity by predicting antigen processing.

The divergence in the generation of negatives and the assumed prevalences may render the comparison in cross-validation metrics with both methods insufficient. Hence, to confirm a better performance against NetH2pan on a dataset, blind testing was implemented from two new H-2K^b^ datasets from IEDB (1034799 and 1035276). Negatives were generated following the protocol mentioned above, disregarding positive sequences that do not have a protein accession or cannot be reframed into 12-mers, and by generating random sequences with an assumed prevalence as described in NetH2pan^15^. Given that NetH2pan considers different epitope lengths and substitutions, binarization was done by considering whether binds were predicted overall for a 12 mer sequence. Even if this size was chosen for an evaluation under equal conditions, it should be noted that NetH2pan predicts better shorter sequences on average (Suppl. Fig. 5). In all binary metrics, the LSTM network achieved improved results (Suppl. Figs. 6 and 7). The reported accuracies for were between 96% and 98%, with up to threefold increases in precision.

Notably, in all cases, positives were better detected than in NetH2pan for 12 mers irrespective of the method used to produce negative sequences. On the whole, our approach detected 259 and NetH2pan 86 of a total of 438 antigens across both datasets. Moreover, an ensemble method joining predictive positives from both methods improved detection to 277 with random negatives and 254 with negative sampling.

### Use case

As a result of MuTect2 calling, 4566 variants were identified. From those, 1085 missense transcripts were obtained from VEP corresponding to 345 genes. These were matched against the results from Cufflinks and submitted for prediction. In the end, our proposed software generated a ranking of putative neoantigens. The 35 top-scoring putative neoepitopes are shown in Table 3. The predictions were matched with the original B16 results from Castle et al.^44^ (Suppl. Table 2). Additionally, we compared the rank given by our proposed algorithm’s softmax score with the relative classification of the 12 mer sequence in NetH2pan^15^ and MHCflurry 2.0^16^, obtained by averaging the scores across all of its possible epitope lengths and mutations. Table 3, thus, establishes an order of preference for both methods. Due to sample size limitations, the haplotype H-2D^b^ of the C57BL/6 model is not analyzed but should also be included in a naïve study.

**Table 3:** Putative neoantigens, shown by sequence and gene symbol, ranked by scores for the H-2K^b^ restricted B16 melanoma model.

Rank	Sequence	Gene	Probability	FPKM	Castle et al.	NetH2pan	MHCflurry 2.0
1	NKVVMEYENLEK	Pnp	1.00	3.04	–	24	22
2	KASGFRYNVLSC	Nr1h2	1.00	0.00	–	1	17
3	SQAWTHPPGVVN	Adar	1.00	0.00	–	88	128
4	TFVYPTIFPLRE	Lrrc28	1.00	0.94	–	10	14
5	DKSYTLPSSLRK	Zic2	1.00	1.83	–	27	28
6	TLAQLTWPLWLE	Hjurp	0.43	0.00	–	26	72
7	VDTNMMGHEHIR	Safb2	0.26	24.20	–	140	150
8	AKTAVNDYFQCN	Stox2	0.25	0.00	–	126	179
9	FIAIYHHASRAI	Tm9sf3	0.21	24.29	**	8	40
10	SGASNTTPHLGF	Tab2	0.20	29.21	–	103	58
11	YSSMRMMKEALQ	Herc6	0.18	10.93	–	38	102
12	TRASVTNFQIVH	Tulp2	0.16	0.00	–	43	16
13	AWGVDGTLAQLE	Pkdcc	0.16	5.50	–	118	134
14	VVLLMDALYLLR	Sirpa	0.14	51.24	–	13	49
15	NVTISNLYEGMM	Hjurp	0.13	0.00	–	6	20
16	ARALWFWAFSLQ	Sfi1	0.09	0.00	–	5	47
17	GASSFREAMRIG	Eno3	0.09	29.01	–	21	112
18	LAAIVGKQVLLG	Rpl13a	0.09	1203.49	*	67	5
19	AYSAHTSENLED	Zfp638	0.09	0.00	–	142	181
20	TVAVLGFILSSA	Commd4	0.09	41.28	–	52	30
21	FQYCLFKICRDV	Pla2g12a	0.08	7.05	–	63	101
22	AISAPCIGSPGC	Hjurp	0.08	0.00	–	227	297
23	HKHLMPTQIIPG	Jmjd1c	0.08	3.42	–	144	106
24	MFGIDGFAAVIN	Pdhx	0.07	10.26	–	56	59
25	YQPRQSVSYEDV	Tasor2	0.06	5.16	–	188	220
26	LCPLESRVPHTL	Hjurp	0.06	0.00	–	218	127
27	QMIVFYLIELLK	Jak2	0.05	6.03	–	2	6
28	AHMYEAVALIKD	Dennd5a	0.05	64.21	–	17	9
29	DRIVHALNTTVP	Ccdc58	0.05	0.00	–	70	108
30	NEVDVQEVTHSA	Dlg4	0.04	9.45	–	289	138
31	LAAIVGKQVLLV	Rpl13a	0.04	1203.49	*	48	2
32	QRNRKLDYSSSE	Bod1l	0.04	3.65	–	282	328
33	HLGCIKKKFLQR	Sfi1	0.04	0.00	–	177	225
34	PPTARMMFSGLA	Wiz	0.03	16.70	–	18	167
35	QEEVFAKHVSNA	Smarcc2	0.03	0.00	–	167	104

The gene expression is quantified as fragments per kilobase million. Neoantigens examined in Castle et al.^44^ are classified by selection for validation (*) and reactivity (**). Ranked classification of the average scores of peptide sequences for a complete 12 mer sequence, considering epitope lengths between 8 and 12, given by NetH2pan and MHCflurry 2.0. The ranking of NetH2pan and MHCflurry 2.0 corresponds to binding affinity and presentation scores, respectively.

From an implementation perspective, NAP-CNB simplifies the overall process in comparison with previous murine pipelines by removing the need of performing variant calling separately. In terms of overall performance, the entire pipeline has an execution time of around ten hours in a local server using two CPU cores. This duration corresponds to steps between preprocessing of the RNA-Seq and quality analysis to affinity prediction. The levels of abundance are presented to guide the user in selecting a candidate.

## Discussion

The proposed pipeline provides an integrated software solution for mouse neoantigen MHC class I discovery from RNA-Seq data. The workflow is based on a streamlined process adapted to the resource-efficient and accessibility requirements of pre-clinical research. Notably, we report an affinity binding estimation model that successfully improves previously reported performance. The B16 case study also shows a good number of putative neoantigens that are coherent with literature estimates^44^. A functional validation measuring T-cell immune responses by ELISPOT or intracellular IFN-gamma staining in mice responding to B16 tumors would be required to validate the prediction results.

In terms of the actual prediction algorithm, the RNN-based approach presents an AUC ROC of 95% in cross-validation. Compared with the current NetH2pan benchmark model^15^, it represents an enhancement in terms of accuracy and precision for the H-2K^b^ haplotype in both cross-validation and blind testing metrics, with a threefold increase of precision in the latter. However, this varies depending on the haplotype used, with H-2K^d^, for instance, lacking such improvements for a blind set. Additionally, this approach eludes a more refined version of immunogenicity prediction as the one presented by MHCflurry 2.0^16^, although it presents a comparable performance in their binding affinity estimation. Thus, these results may reinforce sequential models’ usefulness as an efficient solution to antigen binding prediction against more conventional neural network approaches. Future lines of research may include more recent sequential model innovations. Novel types of sequential architectures in transformers and RNNs, such as BERT^45^ and GORU^46^, could serve as enhancers of overall performance. Also, subsequent work in epitope size should aim to reconcile flexibility, which is compatible with an RNN-based framework, with the generation of empirical negative samples. The web server restricts the haplotype utilized for prediction. Even if cross-prediction between haplotypes K^b^ and K^d^ suggests type-specific modeling is an optimal solution, a pan-specific system is part of the future directions.

Concerning data processing, the use of negative empirical sequences and data augmentation should also be considered to improve affinity estimation. Strategies could include generative models such as Gaussian mixtures or adversarial networks (GAN)^47^. Nonetheless, one of the problems posed by the dataset is its reliance on a binarized predictor which hampers the biological meaning of the results. Another problem is the prevalence dependency of precision and recall. Further work should be done to identify an optimal strategy. Finally, our method is characterized by the employment of window sizes that are above the normative length of an epitope to optimize performance, which may imply that reported antigenic determinants are not sufficient information for prediction. Notwithstanding, this limits the usefulness of the tool for short sequences or evaluating multiple epitope sites for a given sequence, which enhances accuracy in NetH2pan^15^ or MHCflurry^16^. However, as NAP-CNB is intended to be employed in its complete pipeline form, this a trade-off against providing a single and more robust score to the user.

The variant calling process poses further challenges. Our approach has prioritized a procedure that functions solely on RNA-Seq data with a conservative selection of mutations, particularly missense SNV. This neglects a high percentage of variants that produce neoantigens^48^ and increases the mutational uncertainty by not including genomic data from DNA-Seq^21^. Advances should proceed in this direction, albeit prioritizing an exclusive RNA-Seq utilization to retain the tool’s cost-effectiveness, which is essential for our open web service to remain reachable.

## Supplementary Information

Supplementary Information 1.

Supplementary Information 2.
