# Supplementary material for: Predicting MHC I restricted T cell epitopes in mice with NAP-CNB, a novel online tool
Source: Sci Rep. 2021 May 24;11:10780. doi: 10.1038/s41598-021-89927-5 (PMC8144223; doi:10.1038/s41598-021-89927-5)
Supplement: Supplementary file 1 — Supplementary Information 1. [file 41598_2021_89927_MOESM1_ESM.pdf]

Predicting MHC I restricted T cell epitopes in mice with NAP-CNB, a novel online tool

Carlos Wert-Carvajal[1,2,3†], Rubén Sánchez-García[1†], José R Macías[1], Rebeca Sanz-Pamplona[4,5], Almudena Méndez Pérez[1], Ramon Alemany[6], Esteban Veiga[1], Carlos Óscar S. Sorzano[1*] and Arrate Muñoz-Barrutia[2,7*]

[1]Centro Nacional de Biotecnología, Consejo Superior de Investigaciones Científicas, Madrid, 28049, Spain. [2]Departamento de Bioingenieria e Ingenieria Aeroespacial, Universidad Carlos III de Madrid, Leganés, 28911, Spain. [3]Bioengineering Department, Imperial College London, Exhibition Road, London, SW7 2AZ, United Kingdom. [4]Unit of Biomarkers and Susceptibility, Oncology Data Analytics Program (ODAP), Catalan Institute of Oncology (ICO), Oncobell Program, Bellvitge Biomedical Research Institute (IDIBELL), L'Hospitalet de Llobregat, 08908, Spain. [5]Centro De Investigación Biomédica en Red de Epidemiología y Salud Pública (CIBERESP), Madrid, Spain. [6] Procure Program, Catalan Institute of Oncology (ICO), Oncobell Program, Bellvitge Biomedical Research Institute (IDIBELL), L'Hospitalet de Llobregat, 08908, Spain. [7] Instituto de Investigación Sanitaria Gregorio Marañón (IiSGM), Madrid, 28007, Spain.

[*]Correspondence: mamunozb@ing.uc3m.es.

SUPPLEMENTARY MATERIAL

Supplementary Figure 1

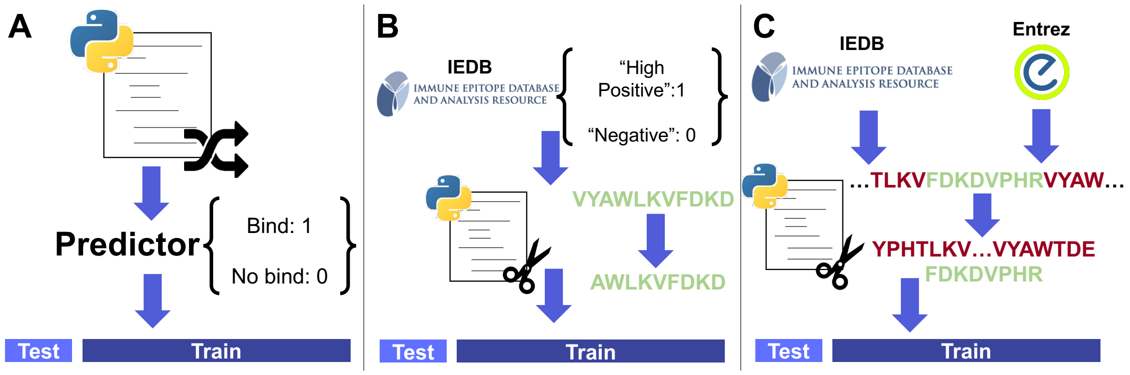

A) Initial architecture configurations used random peptides with the binarized classification from NetH2pan as a toy model. B) For data augmentation and balancing trials, the dataset consisted of epitopes categorized by IEDB as "high positive" and "negative" entries with an equal window. C) In the final model, epitopes were extracted as 12-mers from the original protein, with the rest of the peptide extracted as negatives.

Supplementary Figure 2

A

IMMUNE EPITOPE DATABASE AND ANALYSIS RESOURCE

Epitope	Classification	PDB ID
SIYGFGVTF	Positive-High	P07392
**VTKYYINL**	**Positive**	**P17370**
SDLFPSFVRL	Positive-High	P68609
DVTTFLSM	Positive	P24770

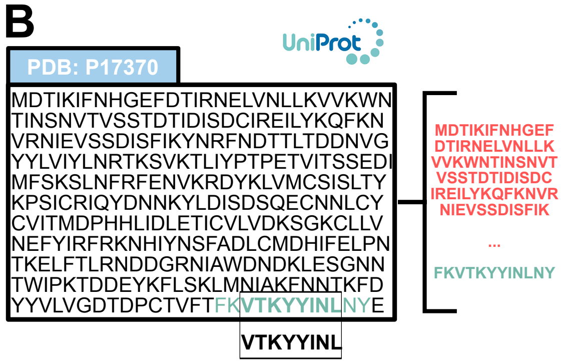

Data from IEDB (A) is aligned through the Smith-Waterman algorithm with the PDB entry from UniProt to obtain an extended sequence and get negatives for training with the remaining sequence. (B) These sequences are then balanced on-batch for different prevalence levels.

Supplementary Figure 3

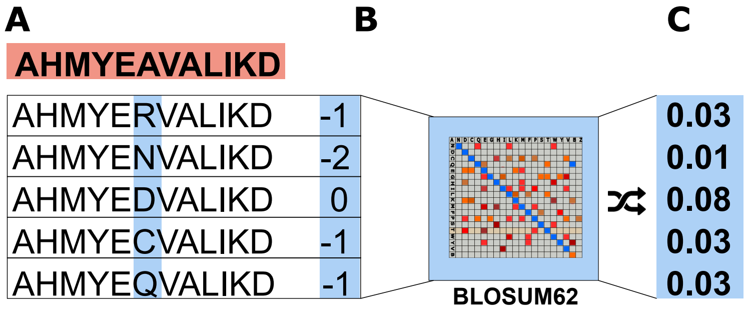

For a sequence (A), data is augmented through mutations at a random location. The new amino acid's similarity score, extracted from the BLOSUM62 matrix (B), normalized using a softmax function (C), is higher than a given tolerance. The

number of amino acids to be mutated and the proportion of sequences per batch to be augmented serve as additional optimization parameters.

Supplementary Table 1

A)

#LSTM	Output	AUC	Precision	ACC	Sensitivity	Specificity	F-1
1	10	0.978	0.994	0.972	0.954	0.993	0.999
2	5	0.999	1.000	0.995	0.997	0.993	0.996
3	5	1.000	0.994	0.984	0.976	0.993	0.985

Tests on different depths in long short-term memory units for a batch size of 10 and 5 epochs. We considered the best results in terms of output size in the range of 5,10, or 20. The training was performed on the toy model (Supplementary Figure 1A). Metrics correspond to a 10% test set.

B)

Embedding	Dim	AUC	Precision	ACC	Sensitivity	Specificity	F-1
One-hot	22	1.000	0.976	0.987	1.000	0.972	0.988
Kidera *et al.* [1]	10	0.999	0.991	0.982	0.976	0.990	0.983
Liu *et al.* [2]	11	0.998	0.991	0.972	0.957	0.990	0.974
Atchley *et al.* [3]	5	0.999	0.973	0.982	0.994	0.969	0.984

Performance metrics of embeddings extracted from literature for amino acid representation. The toy model (Supplementary Figure 1A) was not cross-validated for each case, hence, we used the same hyperparameters for comparison. Metrics correspond to a 10% test set.

C)

On batch additions	Tolerance	AUC	Precision	Sensitivity	Specificity	F-1
0	-	0.937	0.763	0.766	0.943	0.765
1	0.00	0.957	0.767	0.838	0.941	0.801
	0.05	0.947	0.774	0.689	0.952	0.729
	0.10	0.934	0.726	0.796	0.926	0.759
2	0.00	0.943	0.737	0.818	0.931	0.775
	0.05	0.931	0.701	0.718	0.927	0.709
	0.10	0.919	0.668	0.711	0.901	0.689
5	0.00	0.948	0.754	0.826	0.939	0.788
	0.05	0.934	0.756	0.593	0.955	0.665
	0.10	0.933	0.713	0.713	0.931	0.713

Measures from the data augmentation tests for different numbers of new peptide entries per batch at different tolerance thresholds. A batch size of 20 entries and 20 epochs was used. Tolerance denotes the maximum normalized BLOSUM62 similarity for augmentation (Supplementary Figure 3). Thus, only mutations higher than the tolerance score were allowed. Augmentation changes were tested for method validity on the intermediate high-confidence model (Supplementary Figure 1B) with the mean of 5-fold cross-validation shown for each metric. See Additional file 2- Grid search parametrization

Supplementary Figure 4

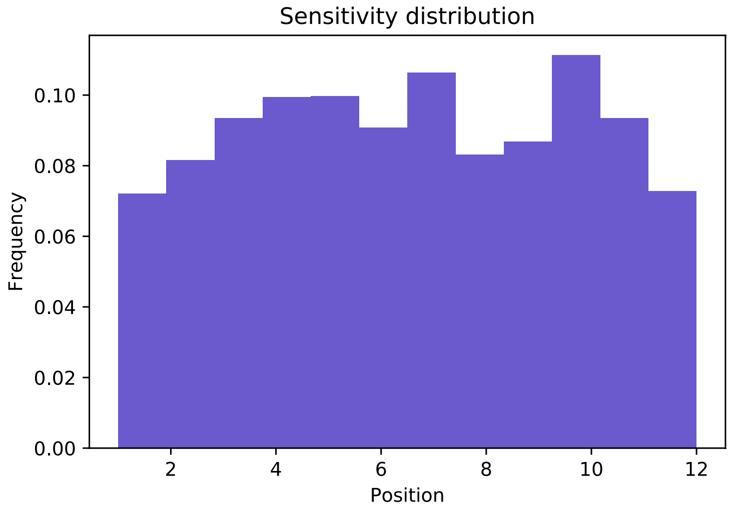

To characterize the susceptibility of each location to change the outcome, we generated 60,000 natural random peptides and produced random substitutions for each position. As a result, 5,843 sequences representing a 9.74% of the entire series, were prone to modify their prediction through a single amino acid variation. Of those, 83.04% altered their label from negative to positive. The resulting histogram failed to pass a two-sided Kolmogorov-Smirnov test for a uniform distribution ($D = 0.097911$, $p < 2.2e-16$), which implies sensitivity is not evenly distributed.

Supplementary Figure 5

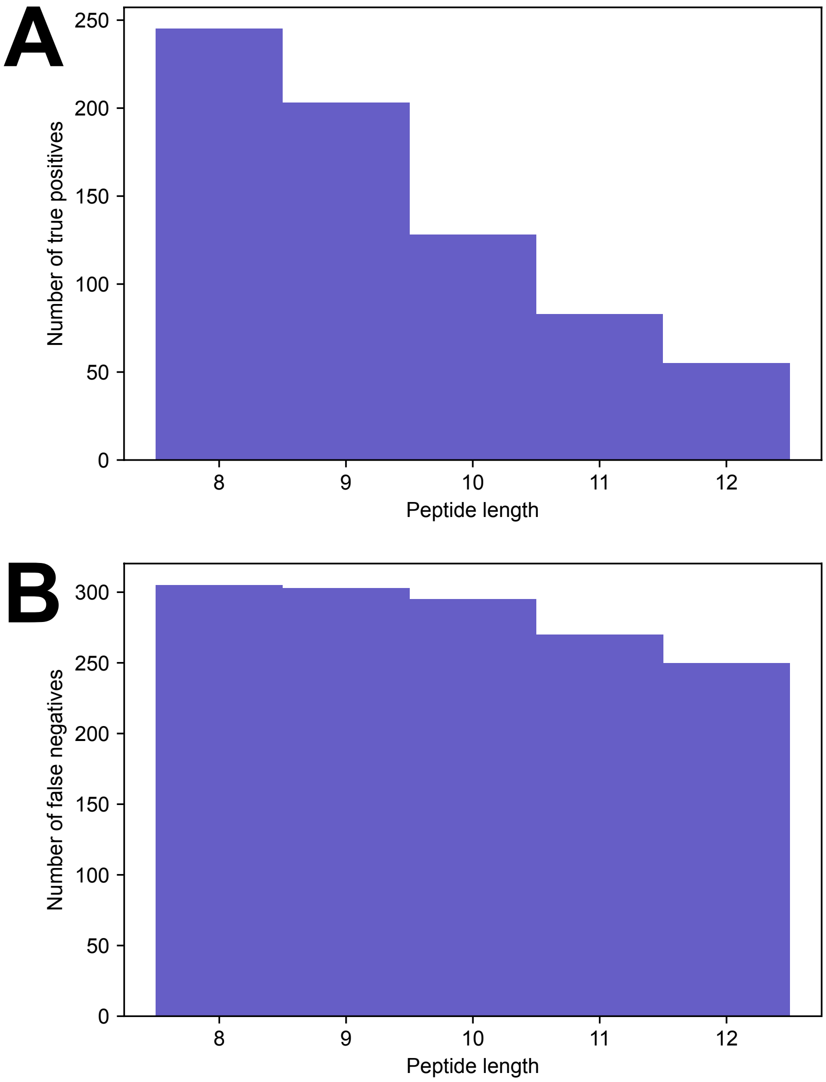

NetH2pan presents a greater sensitivity for short lengths. A) Histogram of the true positives detected at lengths of 8-12. A) Distribution of the false negatives. Sequences were obtained from dataset 1035276 in IEDB and predicted for lengths 8-12. The higher number of true positives and negatives at narrower windows is also due to the overrepresentation of short sequences in the sample (i.e., from a 12mer NetH2pan generates four 8mers), which was corrected by considering a minority rule, in which an arrangement of amino acids was positive or negative if at least one sequence was of that class.

Supplementary Figure 6

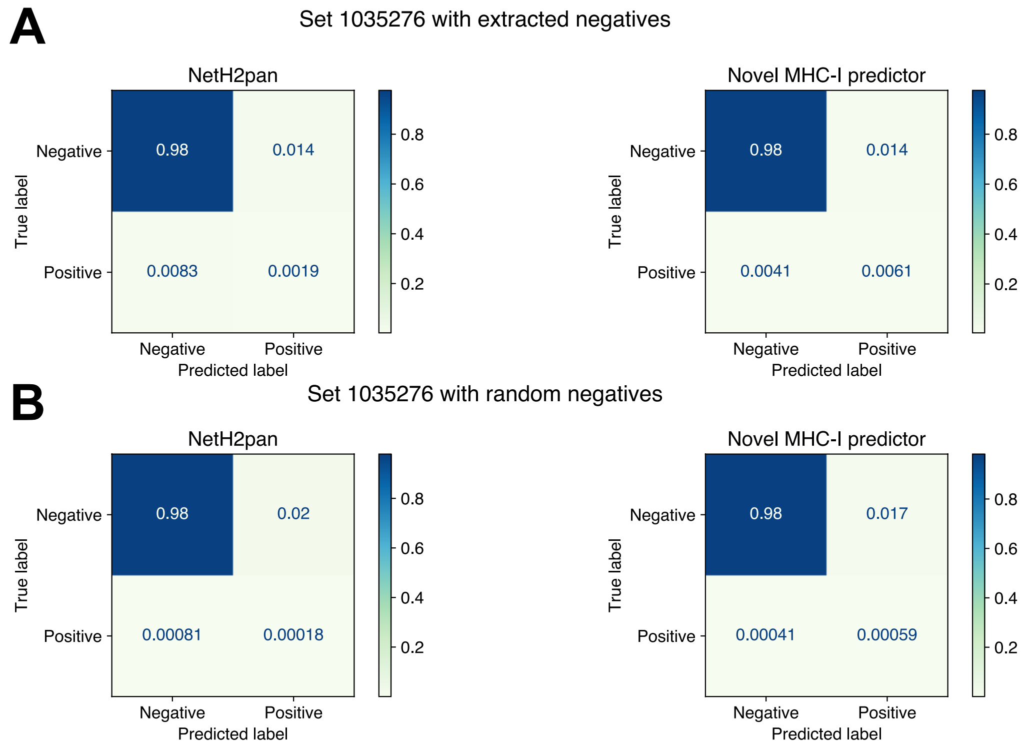

A) Normalized confusion matrix for negatives extracted entries in the 1035276 IEDB dataset. PPV 29.70% (NAP-CNB) and 12.01% (NetH2pan), ACC 98.15% (NAP-CNB) and 97.79% (NetH2pan). B) Negatives obtained from random sequences introduce 999 natural random peptides per positive sequence. PPV 3.33% (NAP-CNB) and 0.89% (NetH2pan), ACC 98.24% (NAP-CNB) and 97.89% (NetH2pan).

Supplementary Figure 7

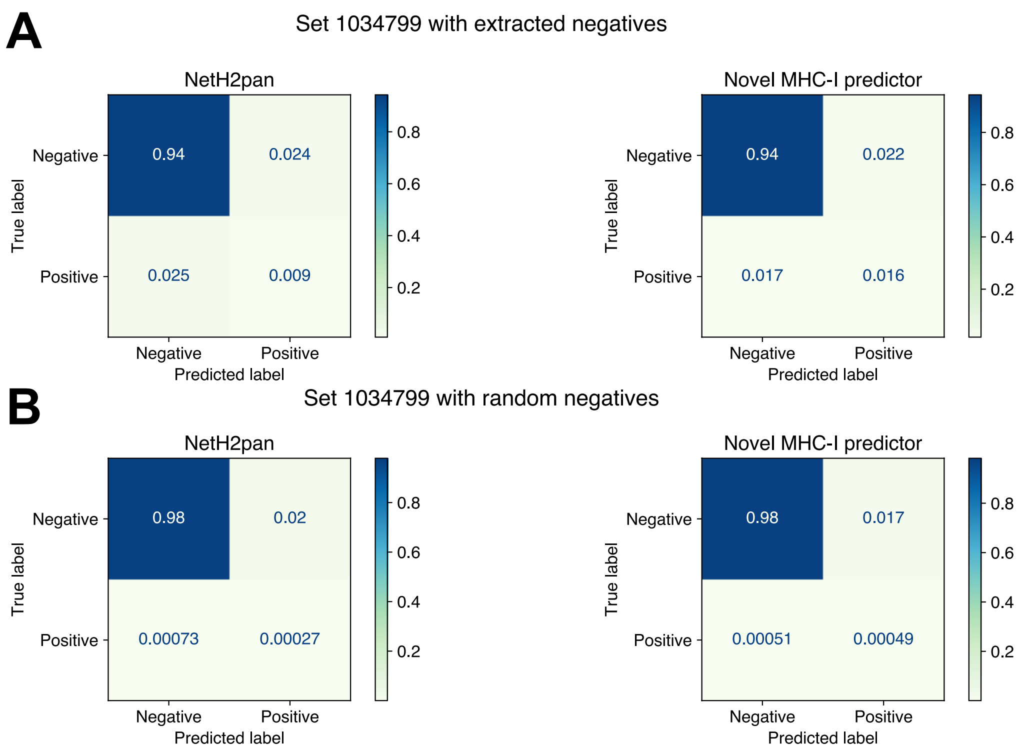

A) Normalized confusion matrix for negatives extracted entries in the 1034799 IEDB dataset. PPV 42.31% (NAP-CNB) and 27.27% (NetH2pan), ACC 96.03% (NAP-CNB) and 95.13% (NetH2pan). B) Negatives obtained from random sequences introduce 999 natural random peptides per positive sequence. PPV 2.81% (NAP-CNB) and 1.30% (NetH2pan), ACC 98.26% (NAP-CNB) and 97.90% (NetH2pan).

Supplementary Table 2

#	Sequence	Gene	Score	FPKM	NetH2pan	MHCflurry 2.0
9	FIAIYHHASRAI	Tm9sf3	0.21	24.29	8	40
55	WYTGEAMDEMEF	Tubb3	0.01	87.0	237	235
64	TQLKKPFLVNNK	Ppp1r7	0.01	8.03	128	31
84	FVDWENVSPELN	Kif18b	0.01	3.32	143	243
158	TTTKKARVSTPK	Dag1	0.0	0.0	259	262
166	QAFIDVMSRETT	Actn4	0.0	87.84	150	245
248	HLNNDVWQIFEN	Plod2	0.0	0.0	204	253
253	GQQLVIQLLHTC	Tnpo3	0.0	39.04	75	90
283	LVLHVVSAAQAE	Sema3b	0.0	31.53	132	226

Complete validated immunogenic mutations from the original paper by Castle *et al.* [4] with the ranking of the mean score given by NetH2pan, MHCflurry 2.0 and the proposed LSTM-based algorithm. Also shown are the fragments per kilobase million (FPKM) of the gene expression. The one-sided Wilconson signed-test

statistic against NetH2pan is of 20 (n.s., $p>0.05$) and 24 with MHCflurry 2.0 (n.s., $p>0.05$).

Supplementary Material for H-2K^d

The total dataset for a window of 12 peptides contained 1,531 positives and 63,686 sequences in total. Using this dataset for prediction with the ANN built for H2Kb, we obtained the binary metrics:

ACC	PPV	Sensitivity	Specificity	F-1
0.964	0.114	0.076	0.986	0.091

It corresponds to a 12mer peptide window with positives obtained from T-cell and MHC ligand assays from IEDB that had a protein entry with the epitope.

Thus, to improve the performance for low positive detections, we trained a different NN for this specific haplotype. Under different network configurations, 8mers systematically outperformed 10mers and 12mers in AUC ROC and PPV in 5-fold cross-validation. The parameters employed for H-2K^b did not produce a good performance after re-training; thus, we use a 5-fold cross-validation routine for optimization.

The dataset used for the 8mer sequences contained 1895 positive sequences and 93281 negative ones.

The cross-validation metrics of the final model were:

AUC ROC (±SD)	ACC (±SD)	PPV (±SD)	Sensitivity (±SD)	Specificity (±SD)	F-1 (±SD)
0.96±0.05	0.982±0.008	0.5±0.2	0.7±0.2	0.987±0.008	0.6±0.2

For the test set, the binary metrics are:

ACC	PPV	Sensitivity	Specificity	F-1
0.974	0.397	0.485	0.984	0.436

Due to its entry date and identification of the original peptides, we identified set 1036855 for blind testing with 9 positives. We generated 92 negatives from our method and 8991 from random negatives.

For this set, our method had less optimal results in overall positives identification. In comparison, NetH2Pan identified one epitope, whereas our approach did not predict any.

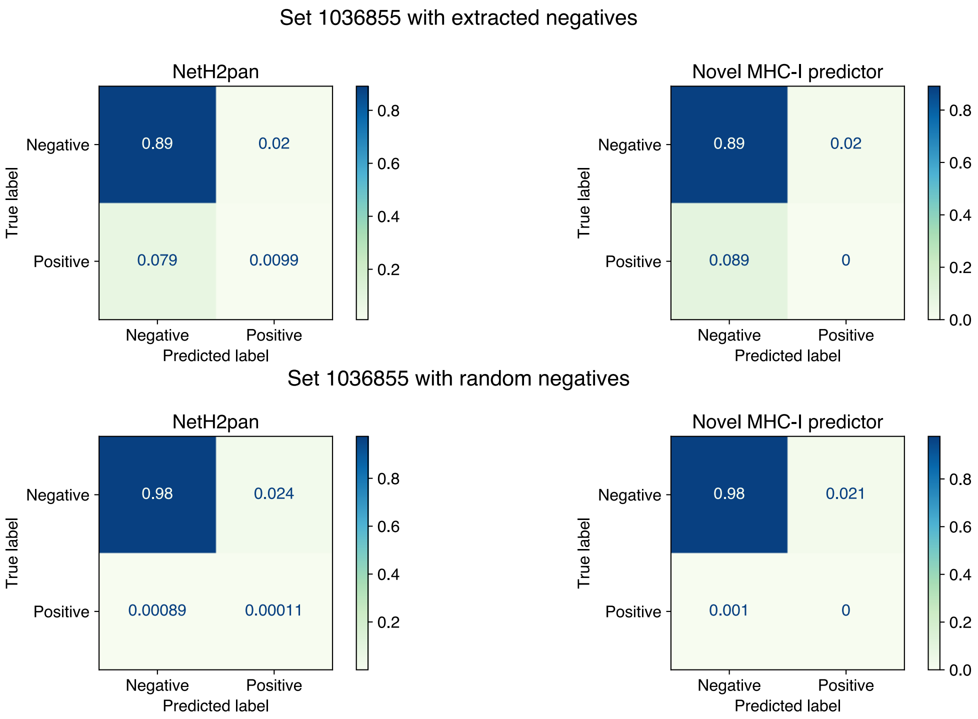

References

[1] A. Kidera, Y. Konishi, M. Oka, T. Ooi, and H. A. Scheraga, "Statistical analysis of the physical properties of the 20 naturally occurring amino acids," *J. Protein Chem.*, vol. 4, no. 1, pp. 23–55, 1985.

[2] W. Liu, X. Meng, Q. Xu, D. R. Flower, and T. Li, "Quantitative prediction of mouse class I MHC peptide binding affinity using support vector machine regression (SVR) models," *BMC Bioinformatics*, vol. 7, no. 1, p. 182, 2006.

[3] W. R. Atchley, J. Zhao, A. D. Fernandes, and T. Druke, "Solving the protein sequence metric problem," *Proc. Natl. Acad. Sci.*, vol. 102, no. 18, pp. 6395–6400, 2005.

[4] J. C. Castle *et al.*, "Exploiting the mutanome for tumor vaccination," *Cancer Res.*, vol. 72, no. 5, pp. 1081–1091, 2012.
